# Hepatic translational rewiring in insulin deficiency identifies Reg3α as an insulin-independent glucoregulatory factor

**DOI:** 10.1210/endocr/bqag100

**Published:** 2026-08-31

**Authors:** Pryscila D. S. Teixeira, Sarah Mathilda Vincent, Romane Meurs, Gloria Ursino, Szabolcs Zahoran, Giulia Lucibello, Marcella M. Authiat, Anne-Claude Gavin, Giorgio Ramadori, Roberto Coppari

**Affiliations:** Department of Cell Physiology and Metabolism, University of Geneva, Geneva 1211, Switzerland; Diabetes Center of the Faculty of Medicine, University of Geneva, Geneva 1211, Switzerland; Department of Cell Physiology and Metabolism, University of Geneva, Geneva 1211, Switzerland; Diabetes Center of the Faculty of Medicine, University of Geneva, Geneva 1211, Switzerland; Center for Integrative Genomics, Génopode, University of Lausanne, Lausanne 1015, Switzerland; Department of Cell Physiology and Metabolism, University of Geneva, Geneva 1211, Switzerland; Diabetes Center of the Faculty of Medicine, University of Geneva, Geneva 1211, Switzerland; Department of Cell Physiology and Metabolism, University of Geneva, Geneva 1211, Switzerland; Diabetes Center of the Faculty of Medicine, University of Geneva, Geneva 1211, Switzerland; Department of Cell Physiology and Metabolism, University of Geneva, Geneva 1211, Switzerland; Diabetes Center of the Faculty of Medicine, University of Geneva, Geneva 1211, Switzerland; Department of Cell Physiology and Metabolism, University of Geneva, Geneva 1211, Switzerland; Department of Cell Physiology and Metabolism, University of Geneva, Geneva 1211, Switzerland; Department of Cell Physiology and Metabolism, University of Geneva, Geneva 1211, Switzerland; Diabetes Center of the Faculty of Medicine, University of Geneva, Geneva 1211, Switzerland; Department of Cell Physiology and Metabolism, University of Geneva, Geneva 1211, Switzerland; Diabetes Center of the Faculty of Medicine, University of Geneva, Geneva 1211, Switzerland

**Keywords:** insulin, diabetes, mRNA translation

## Abstract

**Context:**

Insulin deficiency (ID) causes severe metabolic defects and death if untreated, while insulin therapy does not fully restore metabolic homeostasis. Leptin therapy corrects metabolic abnormalities and promotes survival in rodents unable to produce insulin, suggesting the existence of insulin-independent glucoregulatory mechanisms.

**Objective:**

To characterize hepatic translational remodeling in severe insulin deficiency and determine whether leptin-responsive translational changes can reveal novel glucoregulatory factors.

**Methods:**

We used mice with diphtheria toxin-induced pancreatic β-cell ablation, resulting in severe ID. We assessed hepatic translation by polysome profiling, ribosome profiling (Ribo-seq), and RNA sequencing. To identify leptin-responsive hepatic factors, we compared the hepatic translatome during intracerebroventricular leptin treatment and following leptin withdrawal. Regenerating islet-derived protein 3 alpha (*Reg3α*) was subsequently overexpressed in the liver of ID mice to assess its metabolic effects.

**Results:**

ID suppresses hepatic mechanistic target of rapamycin complex 1 (mTORC1) signaling and global protein synthesis while extensively remodeling the hepatic translatome. Translation of anabolic and glucose-metabolism pathways is reduced, whereas transcripts involved in lipid metabolism are selectively enhanced. Leptin treatment markedly increases hepatic Reg3α translation. Hepatic *Reg3α* overexpression significantly improves hyperglycemia in ID mice without altering insulin-stimulated AKT phosphorylation in key metabolic tissues. These findings identify hepatic translational rewiring as an important feature of ID and *Reg3α* as an insulin-independent glucoregulatory factor.

The hormone insulin is secreted by the pancreatic β cells in response to changes in circulating nutrients, in particular glucose (1-5). The central importance of insulin for normal metabolic homeostasis is underscored by the consequence of insulin deficiency (ID), which arises when β cells are severely dysfunctional or lost such as in type 1 diabetes mellitus, late-stage type 2 diabetes mellitus, or following complete pancreatectomy (4, 6, 7). If untreated, ID is a lethal catabolic condition characterized by hyperglycemia, ketoacidosis, hyperglucagonemia, and many other abnormalities. Tens of millions of individuals are affected by ID worldwide, and this number is projected to increase (8-10). While insulin therapy (the cornerstone approach currently available) converts a lethal disease into a manageable condition, it remains unsatisfactory. Indeed, most insulin-treated ID patients do not reach glycemic targets and are at higher risk of developing life-threatening comorbidities (eg, stroke and heart attack) (11-14). Moreover, owing to its potent glycemia-lowering action, insulin therapy can cause iatrogenic hypoglycemia that can be disabling or even fatal (accounting for ∼4%-10% of deaths in type 1 diabetes mellitus) (15-17). Therefore, due to the large number of people affected by ID and the shortcomings of current treatment, it is of paramount medical and societal importance that more adequate therapy be developed. Efforts to address this need have largely focused on insulin itself. Novel insulin analogues or conjugates have been developed to improve pharmacokinetic profiles (18-20), and advanced delivery systems, including artificial pancreas platforms (21), improve the coupling between insulin administration and circulating glucose levels. β-Cell replacement approaches aiming at restoring endogenous insulin secretion from transplanted donor islets (22) or stem-cell–derived cells (23) have also made progress. Nevertheless, these emerging methods fail to fully restore metabolic regulation and remain associated with considerable limitations (eg, lifelong immunosuppressive therapy), highlighting the need for novel and complementary therapeutic strategies for ID. These complementary approaches can be uncovered by investigating insulin-independent mechanisms permitting life without insulin. One of these mechanisms is triggered by leptin monotherapy, an approach that corrects ID-induced metabolic defects and promotes survival of rodents unable to produce insulin (5, 24-26). The fact that the effects of leptin are mediated by hypothalamic neurons (24, 25, 27-29) and the possibility of developing leptin resistance hampered the translational applicability of leptin therapy itself in this context. However, the possibility of treating ID patients with molecules underlying the beneficial effects of leptin led to the identification of new approaches. In previous work, quantitative proteomic analysis of plasma from leptin-treated ID mice identified S100A9 as a peripheral mediator of leptin action (30). S100A9 exerts potent anti-inflammatory effects and normalizes hyperketonemia and hypertriglyceridemia in ID mice (31-33). However, although S100A9 therapy improves hyperglycemia, it does not recapitulate the hyperglycemia-normalizing action of leptin in ID (24-26, 29, 34-36). These findings indicate that additional mediators of leptin's antidiabetic action remain to be identified.

To address this issue, we undertook an approach focused on hepatic translational regulation. The liver is one of the most translationally active organs and plays a central role in maintaining systemic metabolic homeostasis (37). Notably, under conditions of reduced insulin availability, such as fasting, global hepatic protein synthesis is suppressed whereas the translation of specific metabolic pathways remains selectively maintained. In particular, transcripts involved in gluconeogenesis and ketogenesis escape suppression, thereby sustaining continued hepatic glucose production and energy homeostasis (18, 32, 38, 39). These observations suggest that understanding translational regulation is important for defining how the liver adapts its metabolism during nutrient stress. While the hepatic translatome has been partially characterized in acute fasting (40), the consequences of ID on hepatic messenger RNA (mRNA) translation are unknown. Here, to define global and selective changes in hepatic translation, we combined polysome profiling, ribosome footprinting (Ribo-seq), and transcriptomic analysis in a well-validated mouse model of ID. We examined how ID alters hepatic mRNA translation globally and selectively.

To identify hepatic mediators of leptin treatment, we further examined how leptin reshapes the hepatic translatome in ID. Using this approach, we identified regenerating islet-derived protein 3α (Reg3α, also known as hepatocarcinoma-intestine-pancreas/pancreatitis-associated protein HIP/PAP) as a novel insulin-independent glucoregulatory factor. Although Reg3α has been implicated in protecting β cells from inflammatory and cytokine-induced damage, its role in glucose metabolism in the absence of insulin is unknown (41, 42). Here, we show that increased Reg3α levels markedly reduce hyperglycemia in ID mice via a mechanism independent of insulin receptor signaling. These findings uncover a previously unappreciated layer of hepatic translational regulation in ID and unveil a potential new strategy for improving the treatment of diabetes.

## Materials and methods

### Animal studies

All animal research carried out in this study complies with all relevant ethical regulations of the University of Geneva, within the procedures approved by the animal care and experimentation authorities of the Canton of Geneva, Switzerland (animal protocol Nos. GE112B and GE512A). Mice were maintained with a standard chow diet and water available ad libitum in a light- and temperature-controlled environment with a 12-hour light/dark cycle. Tissues were collected between 3 and 4 Pm corresponding to ZT7 and 8. All the experiments were conducted in male mice (aged 10-12 weeks). *RIP-DTR* mice weighing between 25 and 30 g were used as an insulin-deficient model as previously done (32). Diphtheria toxin (DT, Sigma-Aldrich) was dissolved in sterile 0.9% NaCl and intraperitoneally administered (0.5 μg/kg BW) on experimental day 0, 2, and 4 to induce ID. A healthy control group was injected with saline. Metabolic assessments were performed between 4 and 6 days after the last DT injection. Animals displaying undetectable insulin serum levels were selected as ID animals for the study. For chronic leptin treatment, leptin was intracerebroventricularly (icv) administered with a cannula positioned stereotaxically into the cerebral lateral ventricles connected to a small osmotic pump (ALZET) implanted subcutaneously as previously described (25). Leptin (AF-450-31-1MG, PrepoTech) was dissolved in phosphate-buffered saline (pH = 7.4; Invitrogen) solution. DT animals that underwent 10 days of icv leptin treatment are referred to as “ID; icv-leptin.” DT animals that underwent 8 days of icv leptin treatment and were then withdrawn from leptin administration for the following 2 days are referred to as “ID; icv-leptin-STOP.” Overexpression of *Reg3α* using hydrodynamic tail vein injection (HTVI) in mice was performed based on previous studies (30). Overexpression of *Reg3α* is achieved by using pLIVE vectors (Myrus) that allow expression under the control of the albumin promoter. Each mouse received 50 μg of vector encoding for Reg3α or empty vector as control. These animals are referred to as “ID; pLIVE-*Reg3α*” and “ID; pLIVE.” For acute insulin treatment experiments, mice were intraperitoneally injected with human insulin (5 U/kg) and euthanized 10 minutes after injection. These animals are referred to as “ID; pLIVE-*Reg3α* + 5 U/kg insulin.” On experimental end point day, mice were fasted for 3 hours to avoid confounding effects of food. The animals were euthanized by pentobarbital injection and then the liver, heart, gastrocnemius muscle, perigonadal white adipose tissue, and brown adipose tissue were harvested. The number of mice used in each experiment is mentioned in the corresponding figure legend. Care of mice at University of Geneva was within the procedures approved by animal care and experimentation authorities of the Canton of Geneva, Switzerland.

### Protein extraction and immunoblotting

Mice were euthanized, tissues quickly removed, snap-frozen in liquid nitrogen, and subsequently stored at −80 °C. Proteins were extracted by homogenizing samples in lysis buffer (Tris 20 mM, EDTA 5 mM, and NP-40 1% [v/v]), protease inhibitors (P8340; Sigma-Aldrich) and phosphatase inhibitors (P5726, P0044; Sigma-Aldrich) then resolved by sodium dodecyl sulfate–polyacrylamide gel electrophoresis, and lastly transferred to a nitrocellulose membrane by electroblotting. Membranes were blocked with blocking buffer (927-60003; LICORbio) for 1 hour. Primary antibody incubation was performed overnight at 4 °C. The following antibodies were used: 4E-BP1 (Cell Signaling Technology catalog No. 9644, RRID:AB_2097841; 1:1000), Phospho-4E-BP1 (Cell Signaling Technology catalog No. 2855, RRID:AB_560835; 1:1000), Reg3α (Sino Biological catalog No. catalog No. 50185-RP01, RRID:AB_3753733; 1:1000), phospho-AKT(Ser473) (Cell Signaling Technology catalog No. 4060, RRID:AB_2315049; 1:2000), AKT Cell Signaling Technology catalog No. 2920, RRID:AB_1147620; 1:1000), α-tubulin (Proteintech catalog No. 11224-1-AP, RRID:AB_2210206; 1:1000), and β-actin (abcepta catalog No. AM1829b, RRID:AB_10664137; 1:1000). Membranes were washed with TBS-tween before being incubated with a 1:10 000 secondary antibody solution (LICORbio catalog No. 926-32211, RRID:AB_621843; LICORbio catalog No. 926-68070, RRID:AB_10956588) for 1 hour at room temperature, before detection with the LICOR Odyssey Imaging System.

### Circulating substrates

To avoid postprandial confounding effects, food was removed 3 hours prior measurement. Plasma was obtained by centrifugation of tail vein blood in EDTA-coated tubes (Kent Scientific) (3000*g*; 15 minutes; 4 °C). Circulating glucose and ketone body levels (mmol/L) were assessed using a glucose and ketone meter (Statstrip Xpress, Nova Biomedical) on tail vein blood. Plasmatic insulin (Crystal Chem catalog No. 90080, RRID:AB_2783626) and triglycerides (291-94501, Fujifilm Wako Chemicals Europe GmbH) were measured according to manufacturer's instructions.

### Hepatic lipid extraction

Total lipids were extracted from approximately 30 mg of liver homogenate by using an adapted Bligh and Dyer method (43). Samples were maintained on ice prior to processing and, when necessary, sectioned in phosphate-buffered saline. Homogenization was performed in 2-mL protein LoBind tubes containing 2 prewashed metal beads (rinsed with Milli-Q water until no foaming and air-dried). Each sample was mixed with 100 µL of water and 500 µL of methanol and disrupted using a TissueLyser II (Qiagen) at 30 Hz for 40 seconds. Lysates were transferred to glass tubes (Wheaton), and residual material was recovered by rinsing the original tubes with 1 mL of water, which was pooled with the lysate. Lipids were extracted through 2-step liquid–liquid extractions by sequential addition of methanol, chloroform, and ultrapure water to achieve phase separation. Specifically, 900 µL of water, 2 mL of methanol, and 2.3 mL of chloroform were added, followed by thorough vortexing and centrifugation (2000*g*, 2 minutes, 4 °C). The lower organic phase was collected and subjected to additional washing steps with chloroform and water, including re-extraction of the remaining aqueous phase to maximize lipid recovery. After each addition, samples were vortexed thoroughly and centrifuged under the same conditions using a swinging bucket rotor.

The pooled organic phases were collected, while aqueous phases were discarded, and solvents were evaporated under vacuum at room temperature for 2 hours to obtain dried lipid extracts. Lipid films were stored at −20 °C until further use. For downstream analysis of neutral lipids, dried extracts were resuspended in chloroform:methanol:water (20:9:1, v/v/v), vortexed thoroughly, and centrifuged (2000*g*, 2 minutes, 4 °C). Samples were overlaid with argon to minimize oxidation and stored at −20 °C until analysis.

### Lipid measurement by high-performance thin-layer chromatography

Lipid separation was performed by high-performance thin-layer chromatography as previously described (44). Lipid extracts were spotted on Silica Gel 60 plates (1.05631.0001; Merck) using an automated spotter (CAMAG ATS 4). Plates were prewashed in chloroform/methanol (1:1; v/v) and dried under vacuum prior to use. Lipid standards (cholesterol, cholesteryl ester, triacylglycerol, phosphatidylcholine, phosphatidylethanolamine) and liver extracts resuspended in chloroform/methanol/water (20:9:1; v/v/v) were spotted onto the plates. Separation was achieved using a 2-step solvent system: Plates were first developed in chloroform/methanol/ammonium hydroxide (65:25:4: v/v/v) to a migration distance of 5 cm, air-dried briefly, and subsequently developed in hexane/diethyl ether/acetic acid (80:20:2; v/v/v) to 9 cm. Following chromatographic separation, plates were dried under vacuum for 30 minutes, and the lipids were visualized by charring with copper sulfate/orthophosphoric acid reagent as described previously (45). The solution was freshly prepared by dissolving 2.5 g of CuSO_4_ in 20 mL of filtered water, then mixing with 2.35 mL of 85% orthophosphoric acid and adjusting to 25 mL with water. Plates were immersed in 10 mL of staining solution for 1 minute, excess reagent was removed, and plates were dried under vacuum for 15 minutes. Lipids were then charred at 145 °C for 7.5-9.5 minutes. Plates were imaged under visible light and fluorescence at 488 and 546 nm (ChemiDoc MP; Bio-Rad), and lipid spots were quantified by densitometry using ImageJ (ImageJ distribution) (46).

### Histological analysis

After tissue collection, fresh liver from a subset of animals was fixed in 4% formalin. Formalin-fixed liver samples were dehydrated before being embedded in paraffin blocks. Embedded tissues were sectioned in 5 µm slices, then mounted onto glass slides. After deparaffinization and rehydration, the slides were stained with hematoxylin and eosin. Hepatocyte cross-sectional area (µm^2^) was measured using QuPath (47).

### Ribosome profiling

Ribosome profiling (Ribo-seq) libraries were prepared by the BioCode RNA-to-Proteins Core Facility, Faculty of Medicine, University of Geneva. There were 3 to 5 animals per cohort. Briefly, mouse liver tissues were ground in liquid nitrogen and homogenized in lysis buffer (20 mM Tris pH 7.4, 140 mM KCl, 5 mM MgCl_2_, 1% Triton X-100, 1 mg/mL heparin, 100 µg/mL cycloheximide, 1 mM DTT, 25 U/mL Turbo DNase I, and protease inhibitors). Lysates containing 400 µg of total RNA were treated with RNase I (250 U/mg total RNA) for 45 minutes at 22 °C and monosomes were isolated by size-exclusion chromatography using S-400 spin columns. Ribosome-protected fragments (RPFs) of 25 to 34 nucleotides were size-selected by denaturing polyacrylamide gel electrophoresis, dephosphorylated, and ligated to a preadenylated 3′ adapter. Following ribosomal RNA depletion (RiboCop V2, Lexogen), RPFs were reverse-transcribed, circularized, and polymerase chain reaction–amplified with indexed primers. Library quality was assessed by TapeStation and Qubit fluorometry. Libraries were sequenced on an Illumina NovaSeq 6000 at the iGE3 Genomics Platform, University of Geneva (single-end, 1 × 100 bp, ∼100 million reads per library).

### RNA sequencing (total RNA isolation, library preparation, and sequencing)

Total RNA was isolated from the same tissue lysates used for ribosome profiling. RNA was extracted by adding QIAzol reagent directly to cleared lysate aliquots, followed by purification with the Direct-zol RNA Miniprep Plus kit. RNA integrity was assessed by Bioanalyzer; only samples with an RNA Integrity Number of 8.8 or greater were used for library preparation. Stranded mRNA libraries were prepared and sequenced by the iGE3 genomics platform of the University of Geneva on an Illumina NovaSeq 6000 (single-end, 1 × 100 bp, 60 000 000 reads per library).

### Ribosome profiling quality control, mapping, and analysis

Raw reads were adapter-trimmed and quality-filtered using Cutadapt (v1.18) (48), retaining only trimmed reads of 25 to 34 nt corresponding to the expected ribosome-protected fragment size. Reads were aligned to the Mus musculus genome (GRCm38/mm10) using HISAT2 (v2) (49), and reads mapping to ribosomal RNA loci and repetitive elements (RepeatMasker annotation, GRCm38) were excluded using BEDTools (50). Only primary alignments were retained using SAMtools (51). Filtered reads were subsequently mapped to a canonical Mus musculus transcriptome (Ensembl GRCm38, longest CDS per gene) using Bowtie2 (v2.3.5.1) (52). P-site offsets were estimated per read length using riboWaltz (53) in R, based on metagene profiles at annotated start and stop codons. Data quality was confirmed by verifying 70% or more of P-sites in the zero reading frame across CDS regions, consistent with 3-nucleotide periodicity. P-site counts overlapping CDS regions were quantified and normalized by CDS length and sequencing depth to yield RPKM values. Genes with fewer than 10 raw counts across all samples were excluded. Per-codon occupancy was normalized to mean CDS coverage per transcript to correct for gene-level expression differences, and metagene profiles were generated by binning each CDS into 20 equal segments. Differential A-site occupancy between conditions was assessed using Wilcoxon rank-sum tests across biological replicates, including analyses restricted to the first 60 nucleotides of each CDS to capture initiation-proximal pausing.

### RNA-sequencing mapping (counting) and analysis

Raw single-end reads were trimmed using Cutadapt (v1.18) (48) following the same parameters as those for Ribo-seq. Reads shorter than 15 nucleotides after trimming were discarded. Trimmed reads were aligned to the Mus musculus reference genome (GRCm38/mm10) using HISAT2 (49) with default splice-aware parameters. Mapping quality was assessed using the SAMtools (51) flagstat. Genes with fewer than 10 raw counts in all samples were excluded from the downstream analyses.

### Differential expression analysis and gene set enrichment analysis

Differential expression analysis was performed on Ribo- and RNA-seq datasets independently using DESeq2 (54) in R from raw counts. Genes with fewer than 10 counts across all samples were excluded prior to testing. Differentially expressed genes were defined by a Benjamini-Hochberg–adjusted *P* value less than .05 and log_2_ fold change (FC) greater than or equal to 1. Translational efficiency (TE) was estimated as the ratio of normalized Ribo-seq to RNA-seq counts per gene, and differential TE was assessed using a DESeq2 interaction term model (55). Overrepresentation analysis was performed on differentially expressed gene lists using clusterProfiler (56), with gene sets retrieved from MSigDB (57), including GO Biological Process, KEGG, Reactome, and BioCarta collections. Enrichment was tested by Fisher exact test against a background of all expressed genes, with Benjamini-Hochberg correction applied (false discovery rate < 0.05). Gene Set Enrichment Analysis (GSEA) was performed on the full ranked gene list using fgsea (58). Genes were ranked by sign(log_2_FC), and the same MSigDB collections were tested, excluding gene sets with fewer than 15 or more than 500 members. Normalized enrichment scores (NES) were computed across 1000 permutations, and gene sets with NES greater than 1.5 and a false discovery rate less than 0.25 were considered significantly enriched. GSEA was applied independently to RNA-seq and Ribo-seq ranked lists.

### Statistical analysis

All experimental results are represented as the mean ± SEM. Values representation and statistical analyses were performed using GraphPad Prism software 11 (GraphPad Software Inc). Statistical analyses of non-omics data were performed using 2-tail unpaired *t* test when 2 groups were compared, or one-way analysis of variance followed by a multiple comparison ad hoc test (Tukey post hoc test). Statistical significance was set at *P* less than .05.

## Results

### Insulin deficiency alters hepatic protein translation

**Figure 1:**
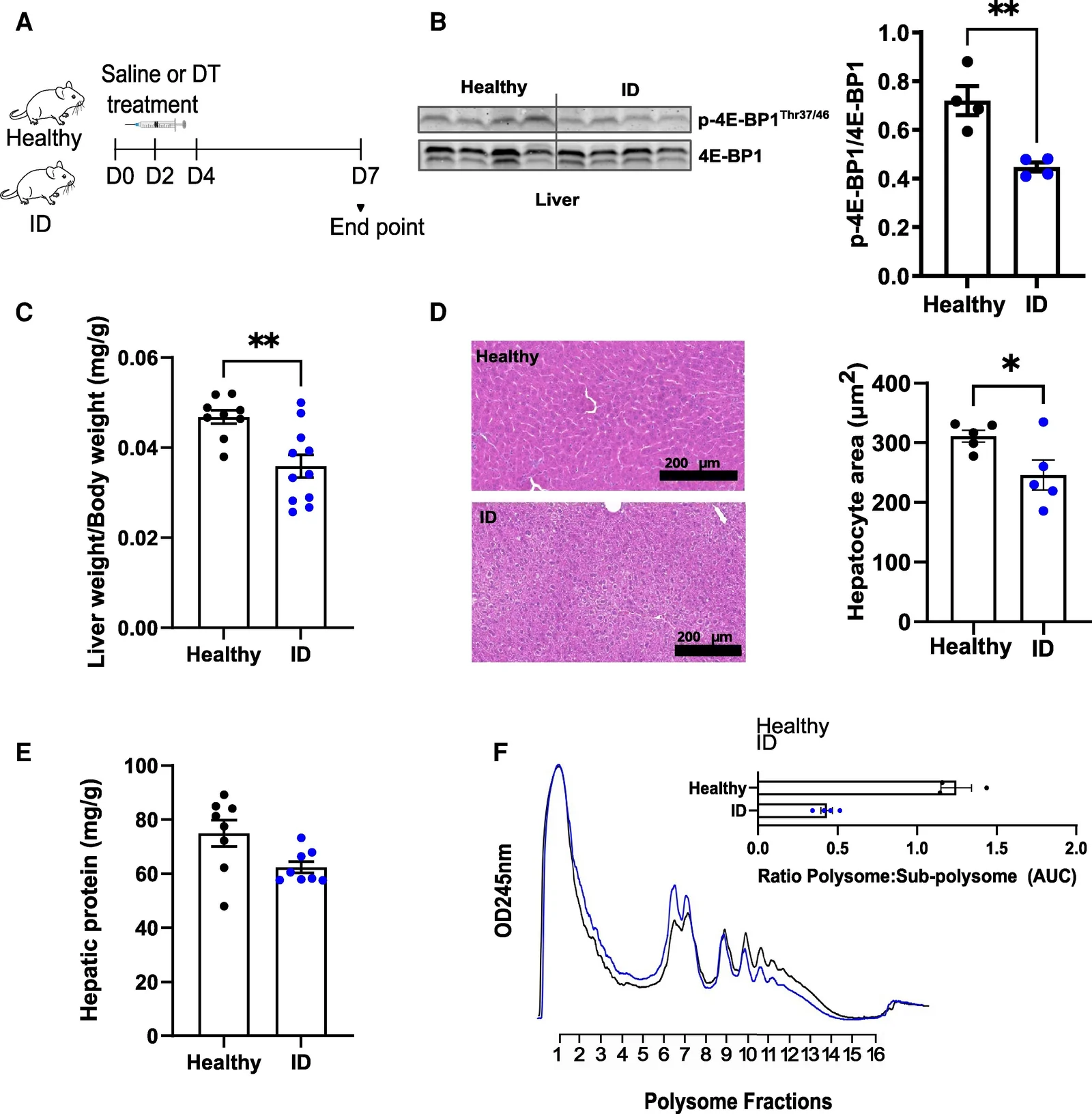
Insulin deficiency (ID) alters hepatic translation. (A) Schematic representation of the treatment protocol of experimental groups. Male RIP-DTR mice aged 10 to 12 weeks were injected intraperitoneally with saline or diphtheria toxin (DT) at day 0 (D0), day 2 (D2), and day 4 (D4). (B) Representative immunoblots of liver lysates from healthy and ID mice at day 7 (D7) and ratio of p-4E-BP1/4E-BP1 protein levels. Measured by densitometry quantification of phosphorylated 4E-BP1 (p-4E-BP1) at Thr37/46 and total 4E-BP1 (n = 4). (C) Percentage liver weight to body weight (n = 9 for healthy, n = 11 for ID). (D) Representative histological images (hematoxylin and eosin staining) of liver sections (scale bar, 200 µm) and hepatocyte quantification of mean area (µm^2^) of healthy and ID mice at D7 (n = 5). (E) Total hepatic protein content (mg/g) (n = 8). (F) Representative polysome profiles of liver samples and histograms showing the relative ratio between polysome:subpolysome fractions (AUC, area under the curve) from healthy and ID mice at D7 (n = 4). Data are represented as mean ± SEM. Statistical analyses were performed using 2-tailed unpaired *t* test;*P < .05; **P < .01.

To address the effect of ID on hepatic mRNA translation, we modeled human ID by using mice carrying the *RIPDTR* allele. This allele encodes the diphtheria toxin receptor (*DTR*) sequence under the control of the rat insulin promoter (*RIP*), enabling selective pancreatic β-cell depletion and consequential ID following DT administration (32). After DT treatment (Fig. 1A), depletion of insulin-producing β cells was achieved. Indeed, DT-treated *RIPDTR* mice (referred to here as ID) had undetectable levels of circulating insulin, reduced body weight, and increased blood glucose levels (which are key symptoms of ID) as compared to their sex- and age-matched saline-treated *RIPDTR* controls (hereafter referred to as healthy) (Supplementary Fig. S1A-S1C) (59). As expected, ID mice had diminished hepatic mTORC1 activity, as indicated by reduced phosphorylation status of 4E-BP1 in the liver of these mice compared to their controls (Fig. 1B). Because mTORC1 regulates protein and lipid metabolism and cellular growth (38, 60), we assessed liver weight, hepatocyte size, and hepatic protein and lipid contents. Data shown in Fig. 1C and Supplementary Fig. S1D (59) indicate that liver weight is significantly lower in ID mice compared to their controls, a defect associated with decreased hepatocyte size (Fig. 1D). Hepatic protein content was significantly reduced in ID mice compared to their controls (Fig. 1E). On the other hand, our results shown in Supplementary Fig. S1E to S1H (59) indicate an accumulation of triglycerides and other classes of lipids such as cholesterol, phosphatidylcholine, and phosphatidylethanolamine in ID mice compared to controls. Altogether, these findings are consistent with major hepatic metabolic rewiring and reduced global protein synthesis in ID.

To investigate the effect of ID on hepatic protein translation, we first assessed hepatic polysome profiles. Separation of hepatic lysates by sucrose gradient ultracentrifugation revealed a marked reduction in polysome abundance in ID mice. Consistent with this, the ratio of polysomes to subpolysomal fractions was significantly lower in ID mice than in healthy controls (Fig. 1F). This shift was accompanied by an accumulation of free mRNAs and/or ribonucleoprotein particles as well as monosomes (Fig. 1F). These data indicate a global impairment of mRNA translation in the liver of ID mice.

**Figure 2:**
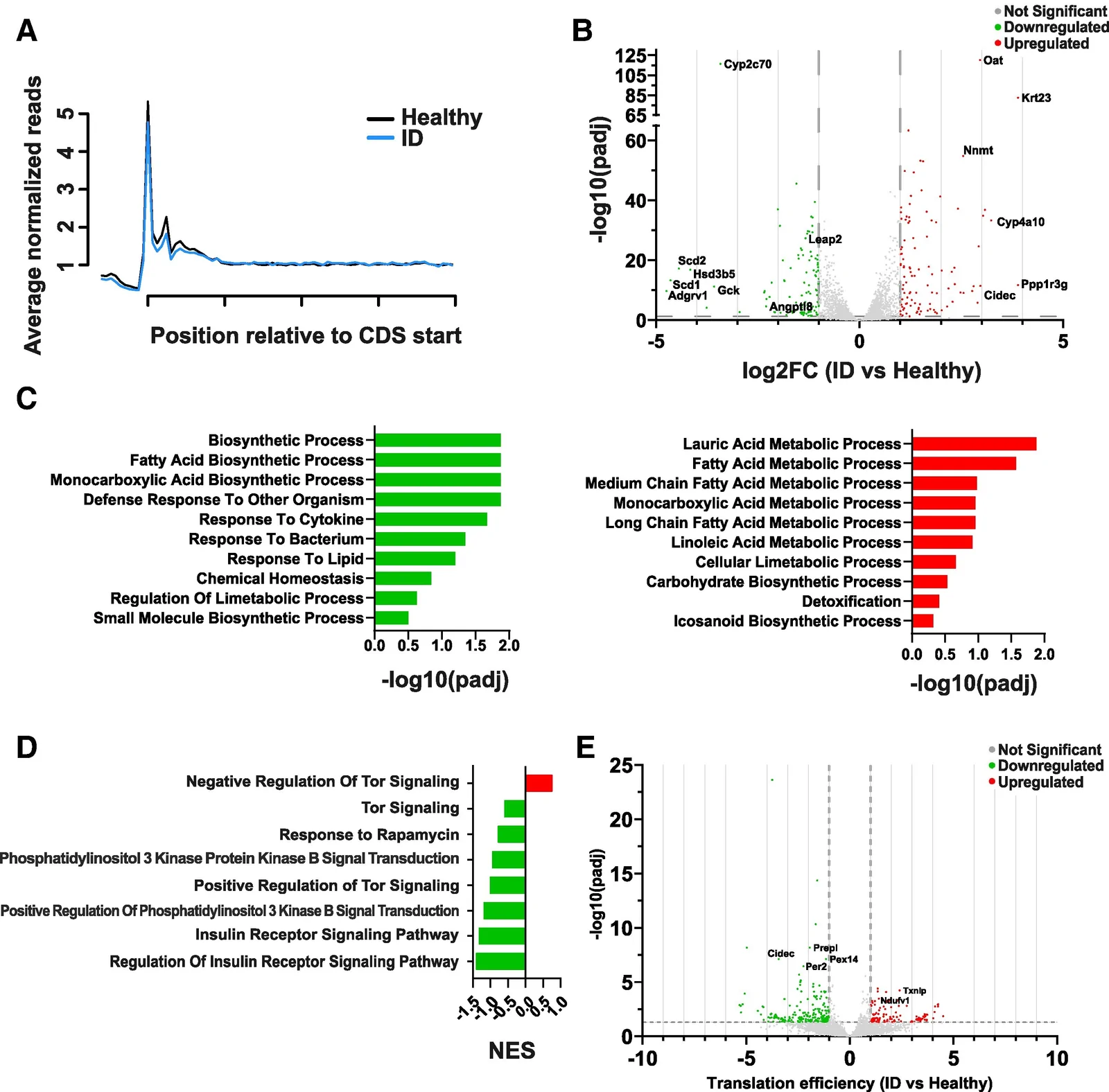
Identification of insulin deficiency (ID) effect on the hepatic translatome. (A) Metagene analysis showing ribosomal protected fragments (RPF) distribution at the 5′ untranslated region up to 200 nucleotides after the start codon of the open reading frames in healthy and ID mice. (B) Volcano plot of genes upregulated and downregulated in the Ribo-seq analysis. (log_2_FC| > 1, adjusted *P*<.05). Selected genes are labeled. (C) Bar chart of top enriched terms for the genes upregulated and downregulated in Ribo-seq of liver of ID mice (GO term, –log10–adjusted *P*). (D) GO terms associated with the mTORC/PI3 K signaling pathway in our gene set enrichment analysis (GO term, normalized enrichment score, NES). (E) Volcano plot of translation efficiency of genes upregulated and downregulated detected by integrative analysis of Ribo-seq and RNA-seq in liver under ID vs healthy conditions. Selected genes are labeled (n = 5).

To further investigate the consequence of ID on hepatic translation, we employed Ribo-seq, a method that provides a direct proxy for the rate of protein synthesis by deep sequencing ribosome-protected mRNA fragments (RPFs) (61). Ribo-seq libraries were prepared from liver lysates of ID and healthy mice and subjected to deep sequencing. RPKM-normalized values were used for pairwise sample-correlation analysis as part of Ribo-seq quality control (Supplementary Fig. S2C) (59). Quality-control analyses of the Ribo-seq data revealed the expected triplet periodicity, with the majority of ribosomal footprint P-sites mapping to frame 0, consistent with correct codon decoding of annotated open reading frames (ORFs) (Supplementary Fig. S2A) (59). Moreover, RPF lengths were within the expected range of 27 to 30 nucleotides (Supplementary Fig. S2B) (59), and pairwise correlation analysis demonstrated high concordance between samples (Supplementary Fig. S2C) (59). Metagene analysis revealed a tendency of global reduction in RPF density within 5′ untranslated regions and across the first approximately 30 nucleotides downstream of annotated start codons in ID liver compared with healthy controls (Fig. 2A). The decrease in ribosome occupancy was not attributable to altered codon usage (Supplementary Fig. S2D) (59) and was followed by a recovery of read density further into the coding sequence, reaching levels comparable to controls (Fig. 2A). These results suggest altered translation initiation and early elongation in ID.

Genes with differentially abundant RPF counts between ID and healthy mice are depicted in the volcano plot shown in Fig. 2B. These data reveal widespread alterations in ribosome engagement across several hepatic mRNAs in ID mice (Fig. 2B). Notably, despite the overall reduction in global translation inferred from polysome profiling (Fig. 1E and 1F), some mRNAs were more highly translated in ID vs healthy conditions (Fig. 2B). In total, 107 transcripts were translationally downregulated and 104 were upregulated in ID livers (Fig. 2B and Supplementary Table S1) (59).

GSEA of ribosome occupancy data uncovered marked divergence between translationally upregulated and downregulated biological processes. Translationally upregulated genes sets were predominantly associated with lipid metabolism, in particular fatty acid and monocarboxylic acid metabolic processes (Fig. 2C). Additional enriched terms included detoxification pathways and eicosanoid biosynthesis, suggesting broader remodeling of hepatic lipid handling and metabolic function. In contrast, translationally downregulated gene sets reflected suppression of lipid biosynthetic and immune response pathways (Fig. 2C). In line with data shown in Fig. 1B, GSEA of Gene Ontology biological process (GO:BP) terms related to mTOR/PI3K signaling revealed predominantly negative enrichment across this pathway (Fig. 2D). Curated pathway terms exhibiting negative NES included TOR signaling, response to rapamycin, insulin receptor signaling pathway, and PI3K-AKT signal transduction. Notably, the only positively enriched term was negative regulation of TOR signaling, further supporting an attenuation of mTOR activity at the translational level. Collectively, these findings indicate a coordinated shift in the hepatic translatome from anabolic to catabolic lipid metabolic programs under ID conditions.

To assess how efficiently individual mRNAs are translated (translatability), we performed differential TE analysis by computing the ratio of RPF abundance to mRNA abundance for each gene and plotted the resulting log_2_FC against statistical significance as a volcano plot (Fig. 2E). Using thresholds of log_2_FC of 1 or greater and adjusted *P* value less than .05 (−log10(*P*adj) ≥ 1.3), we identified 331 translationally dysregulated genes out of 6475 detected genes. Among these, 117 and 214 genes displayed significantly increased or decreased TE in ID vs healthy controls, respectively (see Fig. 2E).

Among genes with reduced TE, several are functionally linked to metabolic regulation. *Cidec*, which is known to enhance triglyceride-rich lipid droplets accumulation in hepatocytes; *Per2*, a core circadian clock gene whose disruption impairs hepatic glucose metabolism and insulin signaling; and *Pex14*, a peroxisomal membrane protein known to stimulate peroxisomal lipid oxidation in hepatocytes, displayed reduced translational efficiency in ID compared with healthy controls. Conversely, the genes with increased TE included *Txnip*, which is known to promote hepatic glucose production while driving triglyceride and lipid accumulation in hepatocytes, and *Ndufv1*, a structural subunit of mitochondrial complex I driving hepatic oxidative phosphorylation whose dysfunction has been associated with the onset and the progression of metabolic dysfunction-associated steatohepatitis, point to the dysregulation of mitochondrial energy metabolism in the liver. These observations indicate that ID affects translational control of metabolic gene networks beyond steady-state mRNA regulation.

Consistent with this notion, ID markedly altered the translation of genes involved in glucose metabolism and hepatic steatosis susceptibility. *Gck,* a key glycolytic enzyme, and *Fabp5*, which participates in fatty acid transport, were among the most strongly downregulated genes, highlighting suppression of hepatocytic glucose utilization and lipid-handling pathways (Fig. 2B). At the transcript abundance level, *Gck* was significantly reduced, while *Fabp5* followed a similar downward trend at both the mRNA and translational levels. Additionally, *Pklr*, encoding the final enzyme of glycolysis, and *Acly*, a key enzyme linking glucose metabolism to lipid synthesis, were downregulated at the mRNA abundance level (Supplementary Fig. S3A) (59).

Conversely, pathway enrichment analysis of translationally upregulated transcripts revealed strong associations with lipid metabolism (Fig. 2C). Several of these transcripts play established roles in lipid storage and lipid droplet dynamics, including *Apoa4*, *Cidec*, *G0s2*, and *Plk3* (see Fig. 2B). Coordinated translational upregulation of these factors may promote hepatic lipid accumulation in ID mice (Fig. 2B and 2C; Supplementary Figs. S3A and S3B) (59). Together, our results indicate that ID reprograms the hepatic translatome through global suppression of anabolic and glucose metabolic pathways, accompanied by selective translational activation of genes involved in lipid metabolism.

### Leptin therapy reshapes hepatic protein translation in insulin deficiency

**Figure 3:**
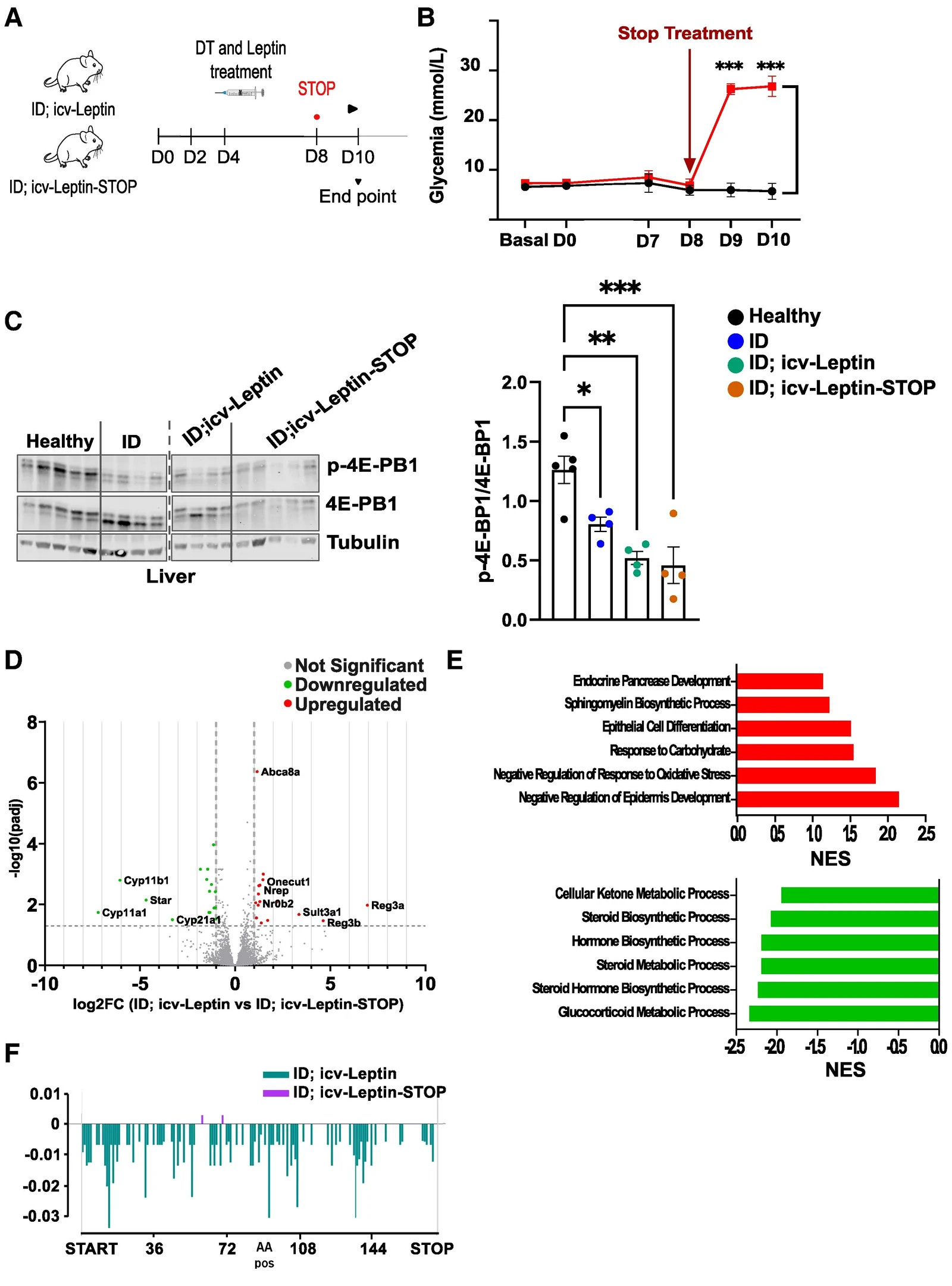
Central leptin reshapes hepatic translatome by increasing translation of genes involved in tissue regeneration, lipid catabolism, and immune response. (A) Schematic representation of the treatment protocol of experimental groups. Male RIP-DTR mice aged 10 to 12 weeks are treated with saline or diphtheria toxin (DT). DT-treated animals receive leptin via intracerebroventricular (icv) infusion from day 0 (D0). ID; icv-leptin group received continuous treatment through D10, while insulin deficiency (ID) icv-leptin-STOP cohort had treatment interrupted at D8 (n = 4-5). (B) Glycemia (mmol/L) levels over treatment and interrupted treatment. (C) Representative immunoblots of liver lysates from healthy, ID, ID; icv-Leptin and ID; icv-Leptin-STOP cohorts at D10 and ratio of p-4E-BP1E/4E-BP1 protein levels. Measured densitometry quantification of phosphorylated 4E-BP1 (p-4EBP1) at Thr37/46 and total 4E-BP1, and α-tubulin (n = 4-5; cropped image at dashed line). (D) Volcano plot of genes upregulated (red) and downregulated (green) in the Ribo-seq analysis comparisons made between ID; icv-leptin vs ID; icv-leptin-STOP (log_2_FC| > 1, adjusted *P* < .05). Selected genes are labeled. (E) Bar chart of top enriched terms for the genes upregulated and downregulated in Ribo-seq of liver of ID mice (GO term, normalized enrichment score, NES). (F) Codon-level ribosome occupancy across the *Reg3α* coding sequence (CDS) in healthy (blue, lower) and ID (purple, upper) mice, derived from Ribo-seq. Each bar represents normalized ribosome density at a given amino acid (AA) position, from the START to the STOP codon. All data are expressed as mean ± SEM. Statistical significance was assessed by one-way analysis of variance with Tukey post hoc test; *P < .05; **P < .01; ***P < .001.

Icv leptin administration improves hyperglycemia, hyperketonemia, hypertriglyceridemia, and other metabolic abnormalities associated with ID in rodents (24, 25). To identify the mechanisms underlying the beneficial actions of leptin, we generated two experimental groups: (i) DT-treated *RIPDTR* mice that underwent icv leptin treatment for 10 days (hereafter referred to as ID; icv-leptin) and (ii) DT-treated *RIPDTR* mice treated with icv leptin for 8 days followed by withdrawal of leptin for the subsequent 2 days (hereafter referred to as ID; icv-leptin-STOP) (Fig. 3A). As previously reported (30), hyperglycemia emerged within 2 days of leptin withdrawal in ID mice (Fig. 3B). Accordingly, on day 10, mice in the ID; icv-leptin-STOP group displayed significantly higher circulating blood glucose levels compared with continuously treated ID; icv-leptin mice, with hyperglycemia already evident on day 9 (see Fig. 3B). We hypothesized that changes in the hepatic translatome may contribute to the re-emergence of hyperglycemia following leptin withdrawal. We first investigated the activity of the mTORC1 pathway by assessing phosphorylation of 4E-BP1. Consistent with earlier observations (Fig. 1B), ID was associated with reduced 4E-BP1 phosphorylation in the liver (Fig. 3C). Notably, both ID; icv-leptin and ID; icv-leptin-STOP mice exhibited similarly reduced levels of phospho-4E-BP1 compared with healthy mice (see Fig. 3C), suggesting that central leptin administration does not control this parameter in the liver of ID mice. To identify translatome changes induced by leptin, we performed Ribo-seq on liver samples obtained from ID; icv-leptin-STOP and ID; icv-leptin mice at day 10. Metagene analysis indicated a good frame distribution of the RPFs with a higher percentage of P-sites in frame 0, consistent with accurate decoding of annotated ORFs (Supplementary Fig. S4A) (59), and pairwise analysis further revealed high concordance between samples (Supplementary Fig. S4B) (59).

DE analysis indicates substantial reprogramming of the hepatic translatome induced by leptin, with 18 transcripts translationally downregulated and 16 upregulated in ID; icv-leptin compared to ID; icv-leptin-STOP mice (Fig. 3D and Supplementary Table S2) (59). Gene ontology and biological process analysis revealed enrichment of pathways suppressed (eg, steroid hormone metabolism) or enhanced (eg, epithelial cell differentiation, response to oxidative stress) in ID; icv-leptin compared to ID; icv-leptin-STOP mice (Fig. 3E). Notably, the mRNA of regenerating islet-derived protein 3α (*Reg3α*) was the most upregulated at the translational level by leptin treatment (Fig. 3D). Ribosome occupancy across the *Reg3α* ORF was greatly increased in the liver of ID; icv-leptin compared to ID; icv-leptin-STOP mice, an effect that was independent of changes in total *Reg3α* mRNA levels (Fig. 3F). These findings highlight *Reg3α* as a leptin-responsive hepatic transcript under ID conditions and warrant further investigation of its potential contribution to leptin-mediated metabolic improvements.

### *Reg3α* improves hyperglycemia in insulin deficiency

**Figure 4:**
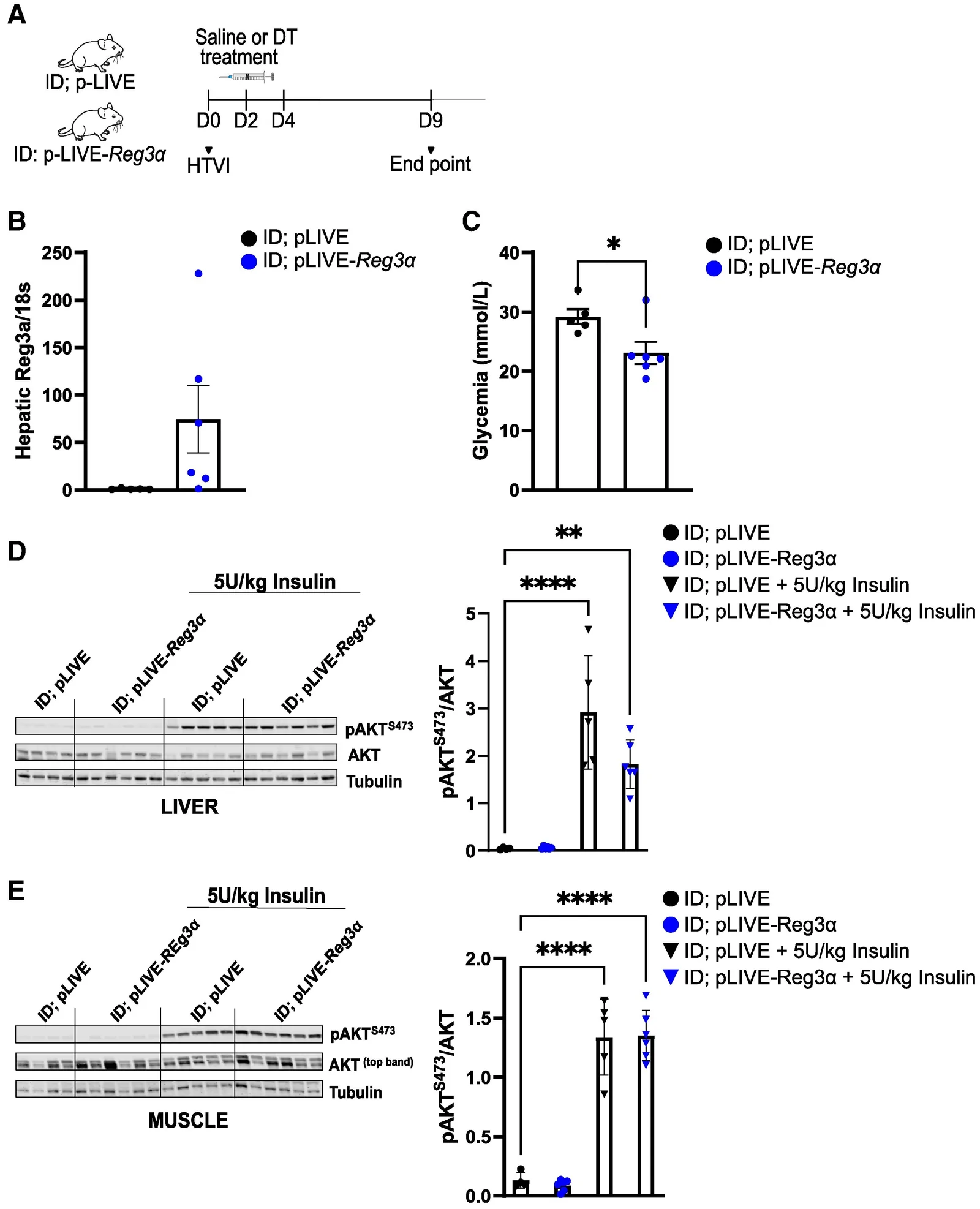
Reg3α improves metabolic control independently of canonical insulin signaling. (A) Schematic representation of the experimental timeline for hepatic overexpression studies. Male RIP-DTR mice aged 10 to 12 weeks were injected intraperitoneally with saline or diptheria toxin (DT) at day 0 (D0), day 2 (D2), and day 4 (D4). Hydrodynamic tail vein injection (HTVI) of pLIVE-*Reg3α*, or empty pLIVE vector was performed simultaneously with the start of insulin deficiency (ID) induction. (B) Hepatic messenger RNA levels of *Reg3α* normalized to 18S ribosomal RNA in ID; pLIVE, and ID; pLIVE-*Reg3α* mice (n = 5-7). (C) Glycemia (mmol/L) levels at D9 (n = 5-6). (D) and (E), Representative immunoblots and densitometric quantification of pAKTS473, total AKT, and tubulin in (D) hepatic tissue, and in (E), gastrocnemius muscle tissue lysates, from ID and ID; pLIVE-*Reg3α* mice, with or without acute insulin challenge (5 U/kg) compared to tubulin. (n = 4-6). All data are expressed as mean ± SEM. Statistical significance was assessed by one-way analysis of variance with Tukey post hoc test or unpaired *t* test as appropriate. *P < .05; **P < .01; ****P < .0001.

To directly test the hypothesis that Reg3α overexpression exerts beneficial actions in ID, we employed HTVI, an approach known to target the liver (62), to deliver plasmids encoding *Reg3α* under the control of the albumin promoter (pLIVE-*Reg3α*) or a control empty vector (pLIVE). HTVI and the first DT administration were performed in *RIPDTR* mice the same day (Fig. 4A). DT-treated *RIPDTR* mice receiving pLIVE (ID; pLIVE) or pLIVE-*Reg3α* (ID; pLIVE-*Reg3α*) displayed comparable levels of severe hypoinsulinemia (Supplementary Fig. S5A) (59). As expected, hepatic *Reg3α* mRNA levels were significantly increased in ID; pLIVE-*Reg3α* mice compared to ID; pLIVE controls (Fig. 4B). These results confirm that both experimental groups were ID and that hepatic Reg3α levels were specifically elevated in ID; pLIVE-*Reg3α* mice.

In ID; pLIVE-*Reg3α* mice, hyperglycemia was significantly reduced compared to ID; pLIVE controls (Fig. 4C); moreover, they showed a trend toward improved circulating triglyceride and β-hydroxybutyrate levels (Supplementary Figs. S5B and S5C) (59). Importantly, these metabolic improvements were not secondary to changes in body weight as this parameter was similar between ID; pLIVE-*Reg3α* and ID; pLIVE mice (Supplementary Fig. S5D) (59).

To determine whether the metabolic effects of Reg3α were mediated through insulin signaling, we assessed insulin-stimulated AKT phosphorylation at serine 473, a canonical downstream marker of insulin receptor activation (63), in multiple metabolically relevant tissues. ID; pLIVE-*Reg3α* and ID; pLIVE mice received an acute intraperitoneal injection of insulin (5 U/kg) or saline, and tissues were collected 10 minutes later. Basal phospho-AKT/AKT ratios were similar in saline-injected ID; pLIVE-*Reg3α* and ID; pLIVE mice in the liver, gastrocnemius muscle, interscapular brown adipose tissue, and perigonadal white adipose tissue (Fig. 4D and 4E; Supplementary Figs. S5E and S5F) (59). As expected, insulin administration increased phospho-AKT/AKT ratios relative to saline in all tissues examined in both ID; pLIVE and ID; pLIVE-*Reg3α* mice (Fig. 4D and 4E; Supplementary Figs. S5D-S5F) (59). However, the magnitude of insulin-induced AKT phosphorylation did not differ between ID; pLIVE and ID; pLIVE-*Reg3α* mice in the tissues analyzed (Fig. 4D and 4E; Supplementary Figs. S5E and S5F) (59). These results demonstrate that overexpression of Reg3α improves hyperglycemia without affecting insulin receptor signaling in ID mice.

## Discussion

Our study identifies translational remodeling as a central feature of the hepatic response to ID. ID emerges as a state of selective translational rewiring in which global hepatic translation and mTORC1 signaling are suppressed (Figs. 1B, 1E, 1F, and 2D). Yet translational output is not uniformly inhibited. Instead, it is redirected toward a restricted set of transcripts, with lipid-metabolism genes prominently represented (Fig. 2C). These data argue that ID also reorganizes hepatic translation, rather than simply repressing it. This interpretation is consistent with the established role of hepatic mTORC1 as a nutrient- and insulin-responsive regulator of anabolic metabolism (18, 38). It is also broadly compatible with observations in fasting, during which hepatic global translational repression coexists with continued translation of adaptive metabolic programs (18, 64). Direct comparison of fasting and ID hepatic translatomes may help resolve which translational responses reflect a general adaptation to reduced anabolic signaling and which instead define a distinct diabetes-associated program, particularly in relation to lipid metabolism.

Among the translational changes induced by ID, several genes showing altered ribosome occupancy provide plausible links to the metabolic phenotype of ID. Reduced ribosome occupancy of Gck may contribute to impaired hepatic glucose utilization (65), whereas increased ribosome occupancy of genes involved in lipid handling and storage, including *G0s2* (66) and *Cidec* (67), may contribute to the hepatic lipid accumulation observed in ID. *Scd1* and *Scd2*, encoding rate-limiting enzymes of monounsaturated fatty acid synthesis (68), were among the most strongly downregulated transcripts at the level of ribosome occupancy, consistent with the broader suppression of fatty acid biosynthetic processes revealed by GO enrichment analysis (see Fig. 2C). In addition, *Leap2* showed markedly reduced ribosome occupancy (see Fig. 2B). As *Leap2* encodes a hepatokine involved in the regulation of food intake and glucose homeostasis (69), its downregulation raises the possibility that reduced LEAP2 activity may contribute to the hyperphagia and hyperglycemia characteristic of ID. Although their causal contribution remains to be established, these factors represent candidate effectors for future mechanistic studies and may reveal potential targets for therapeutic intervention in ID states.

Leptin antidiabetic effect, potentially driven by improved insulin sensitivity, has been observed both in animal models of obesity and type 2 diabetes (5) and, importantly, in humans affected by severe insulin resistance as for example in the context of lipodystrophy (5). Importantly, in severe ID, leptin exerts powerful antidiabetic effects through insulin-independent, largely central nervous system–mediated, mechanisms (24, 25). We therefore examined how central leptin treatment reshapes the hepatic translational state in ID mice. As expected, central leptin administration normalized hyperglycemia in ID mice, whereas leptin withdrawal rapidly reinstated hyperglycemia (see Fig. 3B). Despite this marked metabolic rescue, hepatic mTORC1 activation remained low in leptin-treated ID mice and was comparable to that observed in ID mice that underwent leptin withdrawal (see Fig. 3C), indicating that leptin improved systemic metabolism without affecting hepatic mTORC1. Metagene analysis similarly suggested little recovery of global translation, although our Ribo-seq data revealed substantial selective remodeling of the hepatic translatome in response to leptin (see Figs. 3D and 3E). Together, these findings indicate that metabolic rescue and restoration of the canonical anabolic translation axis are separable events in the context of ID. Our data therefore raise the possibility that leptin does not restore a hepatic healthy state but instead establishes an alternative adaptive state that remains metabolically beneficial despite persistently low mTORC1 activity. In this context, our data refine the current view of leptin action by suggesting that leptin-mediated rescue and mTORC1-dependent anabolic reactivation represent mechanistically distinct layers of hepatic adaptation. They also raise several important questions: Which central nervous system–to-liver signals drive this translational reprogramming, why hepatic mTORC1 remains suppressed despite systemic metabolic rescue, and whether the leptin-rescued liver represents a true return toward homeostasis or instead a distinct metabolically favorable state that remains molecularly altered. Previous studies have indicated that the antidiabetic actions of central leptin in ID are not explained solely by reduced food intake (70), do not require increased peripheral adrenergic activity (71), and do not appear to require hypothalamic-pituitary-adrenal axis suppression (36). Future studies aimed at assessing the contribution of neuroendocrine signals and autonomic outputs on leptin-induced hepatic translational reprogramming are warranted.

Among the leptin-responsive translational changes identified here, *Reg3α* emerged as a particularly compelling candidate. *Reg3α* was not detectable in healthy or ID liver by Ribo-seq analysis; yet it was robustly induced by leptin (see Fig. 3D), suggesting that it is part of a leptin-engaged hepatic program rather than a marker of the diabetic state itself. Indeed, we initially prioritized Reg3α for functional follow-up because previous studies had suggested a potential role for Reg3α in improving glucose metabolism in models of insulin resistance (42). In this context, our data extend that view by showing that hepatic Reg3α improves metabolic control in ID mice through an insulin-independent mechanism. In fact, Reg3α overexpression ameliorated hyperglycemia, hyperketonemia, and hypertriglyceridemia (see Fig. 4C; Supplementary Figs. S5B and S5C) (59), yet did not enhance insulin sensitivity or acute insulin-stimulated AKT phosphorylation in key metabolic tissues (Fig. 4D and 4E; Supplementary Figs. S5E and S5F) (59). The mechanisms underlying these insulin-independent effects remain to be established. Further studies are needed to determine whether Reg3α exerts its glucoregulatory action by engaging insulin-independent pathways affecting endogenous glucose production and/or glucose utilization. Thus, while Reg3α has previously been linked to improved glucose metabolism in insulin-resistant settings, our findings uncover its previously unknown insulin-independent metabolic function. More broadly, these results provide proof of concept that translatome analysis can identify functionally relevant hepatic factors with the potential to mediate the restorative metabolic effects of leptin in ID. At the same time, several key questions remain: whether hepatic *Reg3α* is required for the full metabolic response to leptin, whether its action is liver-autonomous or mediated through secretion, and which downstream signaling pathways underlie its insulin-independent effects. An important next step will also be to determine whether the insulin-independent actions of *Reg3α* can complement insulin therapy, including whether combined treatment reduces insulin requirements, improves glycemic stability, or provides additional metabolic benefits. Such studies will help establish the potential of *Reg3α* as an adjunct to insulin therapy in ID. Importantly, given the central role of the liver in systemic glucose homeostasis, further interrogation of our Ribo-seq dataset may reveal additional candidate effectors and help define the broader hepatic program through which leptin sustains metabolic homeostasis in an insulin-independent manner. Finally, as the present study was conducted exclusively in male mice, whether the hepatic translational responses to ID and leptin, as well as the metabolic effects of Reg3α, are conserved in females remains to be determined.

Taken together, our findings position translational control as a central layer of hepatic adaptation to ID and as a key readout of the metabolic state restored by leptin. Rather than simply mirroring systemic endocrine changes, the hepatic translatome appears to encode distinct pathological and leptin-responsive programs, with *Reg3α* providing proof of principle that functionally relevant effectors can be identified from this layer. More broadly, these results suggest that leptin-induced metabolic recovery may depend not only on correction of systemic metabolism, but also on selective posttranscriptional rewiring within the liver. Answering these questions will be important not only for understanding how leptin reprograms the diabetic liver, but also for establishing whether translational remodeling is itself a tractable therapeutic layer in ID.

## Data Availability

RNA-seq and Ribo-seq data have been deposited at GEO at accession numbers: GSE331248 and GSE331062. Any additional information required is available from the lead contact on request. Supplementary figures (59) and Supplementary tables (Tables S1 and S2) (59) can be found in the Figshare Repository https://doi.org/10.6084/m9.figshare.33265221.

## Abbreviations

DT: diphtheria toxin
FC: fold change
GSEA: Gene Set Enrichment Analysis
HTVI: hydrodynamic tail vein injection
icv: intracerebroventricularly
ID: insulin deficiency
mRNA: messenger RNA
mTORC1: mechanistic target of rapamycin complex 1
NES: normalized enrichment scores
ORFs: open reading frames
*Reg3α*: regenerating islet-derived protein 3 α
Ribo-seq: ribosome profiling
RNA-seq: RNA sequencing
RPFs: ribosome-protected fragments
TE: translational efficiency
